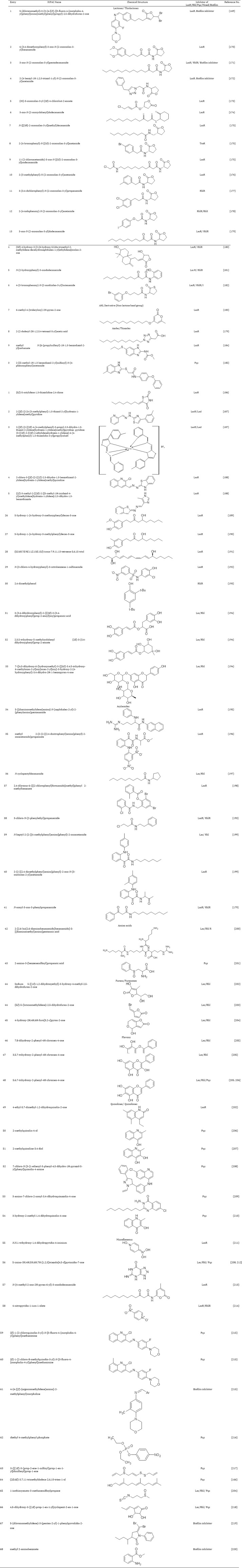# Growing emergence of drug-resistant *Pseudomonas aeruginosa* and attenuation of its virulence using quorum sensing inhibitors: A critical review 

**DOI:** 10.22038/IJBMS.2021.49151.11254

**Published:** 2021-06

**Authors:** Snigdha Bhardwaj, Sonam Bhatia, Shaminder Singh, Francisco Franco Jr

**Affiliations:** 1 Department of Pharmaceutical Science, SHALOM Institute of Health and Allied Sciences, Sam Higginbottom University of Agriculture, Technology and Sciences (SHUATS), Naini, Prayagraj, India; 2 Regional Centre for Biotechnology, NCR Biotech Science Cluster, 3rd Milestone, Faridabad-Gurugram Expressway, Faridabad - 121 001, Haryana, India; 3 Department of Chemistry, De La Salle University, Manila, Metro Manila, Philippines

**Keywords:** Anti-virulence drugs, Biofilm inhibitors, Multidrug resistance, Pseudomonas aeruginosa, Quorum sensing inhibitors

## Abstract

A perilous increase in the number of bacterial infections has led to developing throngs of antibiotics for increasing the quality and expectancy of life. *Pseudomonas aeruginosa* is becoming resistant to all known conventional antimicrobial agents thereby posing a deadly threat to the human population. Nowadays, targeting virulence traits of infectious agents is an alternative approach to antimicrobials that is gaining much popularity to fight antimicrobial resistance. Quorum sensing (QS) involves interspecies communication via a chemical signaling pathway. Under this mechanism, cells work in a concerted manner, communicate with each other with the help of signaling molecules called auto-inducers (AI). The virulence of these strains is driven by genes, whose expression is regulated by AI, which in turn acts as transcriptional activators. Moreover, the problem of antibiotic-resistance in case of infections caused by *P. aeruginosa* becomes more alarming among immune-compromised patients, where the infectious agents easily take over the cellular machinery of the host while hidden in the QS mediated biofilms. Inhibition of the QS circuit of *P. aeruginosa* by targeting various signaling pathways such as LasR, RhlR, Pqs, and QScR transcriptional proteins will help in blocking downstream signal transducers which could result in reducing the bacterial virulence. The anti-virulence agent does not pose an immediate selective pressure on growing bacterium and thus reduces the pathogenicity without harming the target species. Here, we review exclusively, the growing emergence of multi-drug resistant (MDR) *P. aeruginosa* and the critical literature survey of QS inhibitors with their potential application of blocking *P. aeruginosa* infections.

## Introduction

In the last few decades, there has been an alarming increase of reports documented for microbial infections. The mortality caused by pathogenic microorganisms that are currently targeted through known antimicrobials is also a matter of great concern as the microbial populations (bacteria, fungi, viruses, and parasites) have developed strategies to combat antimicrobial drugs worldwide. This has led to an ineffective treatment regime and resulted in the development of resistant strains of microorganisms causing deadly infections. Especially, these resistant microbes have shown fragile access in immune-compromised patients. In this category, *Pseudomonas aeruginosa *is more frequently seen to be associated with healthcare infections (1-3). The versatility of this pathogen to cause several infections is accepted worldwide as it majorly affects aged/immune-compromised patients (elderly and infant patients), HIV patients, individuals undergoing organ transplantation, and people with severe burns and wounds (4, 5). Unlike other bacteria, MDR opportunistic pathogen, *P. aeruginosa, *can grow in niches with high antibiotic pressure as well as may disturb the host-microbiota that may lead to an increase in bacterial virulence or pathogenicity. This causes the bacterium to survive in adverse conditions thus causing high morbidity and mortality due to antibiotic resistance (6, 7). Being a multidrug-resistant strain, *P. aeruginosa* is becoming more difficult to eradicate. The resistance acquired by this pathogen towards several antibiotics is majorly mediated through two types of mechanisms (8). The first mechanism involves a transfer of plasmid among bacteria carrying genes that express β-lactamases or aminoglycosides modifying enzymes (9). The second mechanism involves the mutation in the bacterial genome that causes a targeted mutation in the pathogen. For instance, the gyrase gene present in bacterial membranes is responsible for quinolone resistance due to variable expression of transport protein (regulation of efflux pumps and porins) (10). In both cases, the pathogen develops resistance against the antibiotics that affect bacterial growth; subsequently, bacteria develop their community for enhanced survival which serves as a shield reducing the antimicrobial compound’s activity. For this reason, a different approach is needed to be developed for blocking *P. aeruginosa* infections without interfering with the growth cycle of the pathogen. In recent times, the scientific community identified a novel and efficient strategy called “anti-virulence strategy” that focuses on the inhibition of expression of virulence factors that causes acute and chronic infections, without killing a pathogen (11-13). This non-killing approach renders a low rate of resistance as the survival of pathogens will not be affected by the active drug itself. Thus, in turn, the bacterial community would not be increased to inactivate the active drugs (14, 15). There are several review articles in the literature which are based on QS in *P. aeruginosa *and its associated virulence. Recently, a study (2019) highlighted the importance of *P. aeruginosa *biofilm and its relationship with QS (16). Similarly, another study (2017) described the importance of bacterial QS that can be targeted to modulate virulence among pathogens (17). Along similar lines, a study (2017) also supports the role played by QS in *P. aeruginosa* virulence (18). On the other hand, recently a review (2019) highlighted the importance of naturally derived quorum sensing inhibitors (QSIs) in blocking different signaling pathways in *P. aeruginosa *(19)*.* A systematic review of the various signaling pathways and QS regulators in *P. aeruginosa *was published by Venturi (2006) (20).

We have performed an in-depth literature survey on the QS process in *P. aeruginosa *and its regulation. In this review, we have compiled the data based on various statistical reports published on the growing emergence of resistance in *P. aeruginosa *among clinical samples in varied timelines. This study will help in understanding how resistance develops in this organism for different categories of antibiotics. In addition to this, the current scenario of resistance patterns is alarming and reflects the dire need to develop anti-pseudomonal drugs. We also tried to review and gather the literature on the investigated QSI compounds (chemical and natural origin) targeting LasR, RhL, Pqs, and biofilms to mitigate *P. aeruginosa *infections in an alternative way. This review will help the researchers working from the biological or chemical point of view to understand the growing prevalence of antibiotic-resistant *P. aeruginosa *and the ways to curb these resistant strains by a process of QS inhibition. It will indeed help clinicians and public health professionals to improve their knowledge of the sensitivity or susceptibility of different antibiotics against resistant strains of *P. aeruginosa.*

**Prevalence of drug-resistant of** ***P. aeruginosa***

*P. aeruginosa *is an opportunistic microorganism that causes infection among ill patients, immune-compromised patients, patients compromised by age (infant and elderly patients). Data from the National Nosocomial Infections Surveillance System from 1986-2003 reported *P. aeruginosa* as the second most common cause of pneumonia (18.1%), the third most common cause of urinary tract infection (16.3%), and the eighth-most frequently isolated pathogen from the bloodstream (3.4%) (21). While the overall proportion of infections caused by *P. aeruginosa* remained stable during the 1986-2003 period, however, the proportion of resistant isolates had shown an alarming increase in the consequent years. *P. aeruginosa *resistance to imipenem, quinolones, and third-generation cephalosporins increased by 15.0, 9.0, and 20.0%, respectively. Similarly, a national surveillance study of intensive care unit (ICU) patients from 1993 to 2002, reported a significant increase in multidrug-resistance towards at least three to four agents like imipenem, ceftazidime, ciprofloxacin, and tobramycin. These infections are often problematic, life-threatening, and cause a large number of deaths because of their inherent ability to resist all classes of antimicrobial agents (22, 23). A study was conducted at National Taiwan University Hospital (NTUH) in 2006 where the PDR (pan drug-resistant) strain of *P. aeruginosa *was isolated, this strain has shown resistance to all effective antimicrobial agents including cefepime, ceftazidime, imipenem, meropenem, piperacillin-tazobactam, ciprofloxacin, and levofloxacin leading to resistance of *P. aeruginosa *to all commercially available antimicrobial agents in Taiwan (24). Five years after this report, a national survey on infectious-diseases was conducted by the Infectious Disease Society of America (IDSA), Emerging Infections Network, in 2011, where it was found that more than 60.0% of participants are reported to have infections with a pan-resistant infectious agent, which is untreatable. Many public health organizations have already declared that the human population will face the “catastrophic consequences” of the antibiotic resistance era which will cause havoc for the human civilization (25, 26). Various global organizations like the Centers for Disease Control and Prevention (CDC), IDSA, World Economic Forum, and the World Health Organization (WHO) have announced antibiotic resistance to be a global public health concern (6).

Researchers (2012) observed that the prevalence of *P. aeruginosa* resistant isolates to antimicrobials has increased considerably and the resistance rate of *P. aeruginosa* to antimicrobials such as amikacin, ceftazidime, cefepime, imipenem, and ciprofloxacin was found to be 53.3%, 43.3%, 40.0%, 40.0%, and 33.3%, respectively (27). A study reported in 2014, describes the following resistance rates to cefepime 64.8%, piperacillin 45%, ciprofloxacin 38.9%, levofloxacin 36.1%, gentamicin 37.3%, and amikacin 30% (28). In 2014, EARSS reports showed a high percentage of resistance in *P. aeruginosa *in eastern and southern parts of Europe especially in Germany, Hungary, and Slovakia (29). In 2015, another study conducted for three years from 2013 to 2015, examined *P. aeruginosa* isolates against various antimicrobial agents and reported increasing resistance to a variety of antibiotics, including third and fourth generation cephalosporins such as ceftazidime and cefepime, respectively. A high level of resistance has been reported to β-lactam antibiotics in the United States, Europe, and South America. In the research period, resistance developed by cefepime was significantly increased each year, i.e. 31.6% in 2013, 44.2% in 2014, and 64.5% in 2015, whereas observed resistance to ceftazidime was 59.8% in 2013, 37.3% in 2014, and 42.0% in 2015. The difference in resistance rate towards antimicrobials usually relates to the frequency of use and prescribing practices of hospitals (30). As reported in a study in 2015 on the prevalence of antibiotic resistance among the* P. aeruginosa *population, statistics have shown that the highest resistance is produced against quinolones including ofloxacin (61.3%), ciprofloxacin (60.0%), and levofloxacin (56.4%). Secondly, the aminoglycosides class of compounds (e.g., amikacin and gentamicin) have shown higher rates of resistance to *P. aeruginosa *(31)*. *In 2016, a study was conducted on patients admitted to the ICU of the Tertiary Care Hospital in eastern India for one year (2012-13). The prevalence found among patients to develop nosocomial infections was 24.3% where UTIs were predominant in patients followed by respiratory tract and skin infections (32). A study (2016) reported low to moderate rates of drug resistance to commonly used anti-pseudomonal drugs in *P. aeruginosa* isolates ranging from 4.9% to 30.6%.* P. aeruginosa* showed resistance towards piperacillin-tazobactam, ticarcillin, imipenem, cefepime, amikacin, and meropenem with a prevalence of 4.9%, 22.3%, 19%, 8.3%, 7.4%, and 30.6%, respectively, irrespective of the site of infection. The prevalence of multidrug resistance was 10.7% (33). A study (2017) reflected an increased percentage of drug-resistance in *P. aeruginosa* in patients with community-acquired pneumonia (CAP) (34).

A study by Lila *et al.* (2017) showed an increase of *P. aeruginosa* carbapenem resistance from 2013 to 2015 for imipenem (25.2% in 2013, 26.5% in 2014, and 37.7% in 2015) and meropenem (20.1% in 2013, 23.4% in 2014, and 38.3% in 2015) (35). Similarly, increased rates of imipenem resistance among *P. aeruginosa *(10.2% in 2013, 31.6% in 2014, and 22.1% in 2015) were reported in Croatia, studied by Barsic *et al.* (2004) (36). Benie *et al.* (2017) evaluated *P. aeruginosa* multidrug-resistant (PAMDR) contaminating animal products. All strains of P.aeruginosa isolated from bovine meat, fresh and smoked fish expressed resistance to almost all antibiotics. The prevalence of P.aeruginosa multidrug-resistant was 47.8%, 33.1%, and 20.0%, respectively, in bovine meat, fresh fish, and smoked fish. The percentage of resistance showed by *P. aeruginosa* strains was 98.4% for aztreonam, 51.4% ticarcillin-clavulanic acid, 50.4% ticarcillin, 31.4% piperacillin, 33.6% ciprofloxacin, 17.0% cefepime, 6.9% ceftazidim 7.2% imipenem, 4.5% colistin and 0.0% fosfomycin (37). In 2017, the Government of India declared *P. aeruginosa *as one of the most important pathogens in National Programme for the Containment of Antimicrobial Resistance (5 Year plan, 2012-2017) under National Centre for Disease control. In 2017, WHO published a list of pathogens in which carbapenem-resistant *P. aeruginosa *stands at the second position as critical pathogens. Among different anti-pseudomonas drugs tested, interquartile range showed that almost all are highly susceptible to colistin (96.25-100) whereas less susceptible to gentamicin (24-46.5), ceftazidime (31-55), and cefepime (26-58.75). Under Carbapenems such as imipenem (43-72.5) and meropenem (33-69) interquartile range was observed which were found moderately susceptible (38-43). In 2018, another investigation was conducted by Lila *et al.* (2018) on *P. aeruginosa* isolates at the University Clinical Center of Kosovo (UCCK) using pulse-field gel electrophoresis (PEGE) for identification of anti-microbial susceptibility. The level of resistance was found to be lowest for carbapenems and highest for aminoglycosides. The results exhibited a high sensitivity of amikacin (52.7%), gentamicin (56.6%), and tobramycin (54.5%) towards *P. aeruginosa.* In the same study, piperacillin-tazobactam showed resistance ranges from 26.6% to 44.1% (44). Andrea *et al.* (2019) observed the prevalence and antibiotic resistance profiles of *P. aeruginosa.* The samples were isolated from healthy captive ophidians and also correlated the statistical associations with farming conditions. From this study, the prevalence of multidrug-resistant *P. aeruginosa *strains, as well as strains isolated from young samples and adult samples, were found to be 35.5% and 59.9% respectively where widespread resistance has been observed for cephalosporins, polymyxins, and sulfonamides (45).

**Pathogenicity and virulence of** ***P. aeruginosa***

***Microbiology***

**Figure 1:**
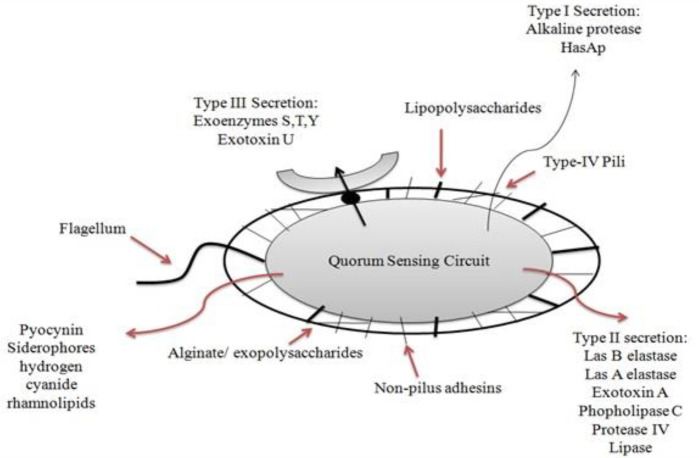
Morphology of *Pseudomonas aeruginosa* representing cell-associated and extracellular virulence factors

*P. aeruginosa *is a Gram-negative, non-fermentative, rod-shaped bacterium, a member of the γ-subdivision of the *Proteobacteria *(26)*. P. aeruginosa *cells measure 0.5 to 1.0 μM by 3 to 4 μM. They are motile due to the presence of one or two polar flagella, grow on a wide variety of culture media over a wide range of temperatures ranging from 0–42 °C. The optimal temperature required for growth is 37 °C, which is also the normal human body temperature. It is a strict aerobe but can grow anaerobically in a nitrate-rich medium. It forms colonies that appear colored according to the pigment overproduced like the production of pigments a) pyocyanin, responsible for bluish-green, b) fluorescein, responsible for greenish-yellow color, and c) phenazine, a yellow color water-soluble pigment (46). It has been recognized as a ubiquitous organism because of its extremely ordinary survival and adaptation abilities in a wide array of environmental conditions. As an opportunistic human pathogen, *P. aeruginosa *has a remarkable capacity to cause diseases in susceptible hosts. It is the major colonizing microbial pathogen for cystic fibrosis (CF) patients and a common infectious agent in nosocomial infections, in infections of patients with severe burns, cancer, transplantation, AIDS, and other immuno-compromising conditions. *P. aeruginosa *is also noted for its conversion from non-mucoid (environmental) to mucoid (clinical) phenotype and its resistance to various antibiotics. *P. aeruginosa *has been found to cause a variety of infections in clinical practice besides chronic CF lung infection, including common acute septicemia from a burn or surgical wound infection, urinary tract infection, corneal ulceration (from wearing contact lenses), endocarditis (caused by intravenous drug use, etc.), and pneumonia (from use of a ventilator and endotracheal tube) (47-49). The morphology of *P. aeruginosa* is diagrammatically represented in Figure 1. 

***Epidemiology: nosocomial infections caused by P. aeruginosa***

*P. aeruginosa *is a common cause of hospitalized infections in immune-compromised patients. The major source of infection is medical equipment and cross colonization from other patients. This bacterium contaminates the medical devices and forms biofilm which poses a serious problem to the patients. For instance, patients may develop catheter-associated urinary tract infections (CAUTIs) as *P. aeruginosa *forms a biofilm on the facets of indwelling catheters. The colonial pathogen causes direct damage to the host tissue and increases the bacterium’s competitiveness (50, 51). *P. aeruginosa *can infect frequently the respiratory tract, blood cells, urinary tract, ear, and soft skin tissues. However, eyes, heart, CNS, bones, and joints are among the sites where the chances of infection are rare. The main cause of infection at these sites is due to trauma following surgery or by the over usage of a drug or any other thing that makes the tissue vulnerable at an immune-compromised state (52, 53). 

*Hospital-acquired pneumonia*

Hospital-acquired pneumonia is the most common life-threatening infection majorly associated with mechanical ventilation and secondly with ICU in hospitals. Ventilator-associated pneumonia (VAP) usually occurs in patients who stay on ventilators for more than 48 hr causing a significant increase in the duration of stay in hospitals cost and death rate. VAP caused by *P. aeruginosa* is associated with trachea-bronchial colonization which is very difficult to eradicate with conventional antibiotics due to the involvement of complex genes in drug resistance, which leads to higher case fatality rates (54-58). *P. aeruginosa *is also considered to be a major cause of permanent blocking of the airways of CF patients, which results in recurrence of lung infections and also decrease in lung function, increasing morbidity and mortality rates (59-61). The infection is mainly associated with a genetic mutation in a protein namely cystic fibrosis transmembrane conductance regulator (CFTR). CFTR is a chloride channel that maintains homeostasis in epithelial cells. The disruption in the regulation of chloride ion transport across membrane results in impaired mucociliary clearance due to an increase in sodium absorption, causing obstruction and mucus hypoxia hence supports colonization of *P. aeruginosa*. Patients with chronic obstructive pulmonary disease (COPD) are also susceptible to respiratory tract infection by *P. aeruginosa *and show similar symptoms to CF patients (62-65). In addition, various studies reported that *P. aeruginosa* produces Pyoverdine (a siderophore, ion-chelating molecule) (66, 67) which functions as a signal molecule since it persuades the expression of virulence and biofilm formation causing chronic lung infections in patients with CF (68, 69). 

*Blood infections*

Although very few studies reported different sources of infection for bloodstream infections (BSI) with* P. aeruginosa*, it is considered to be a serious life-threatening condition and a major cause of the increased rate of morbidity and mortality, as the incidence of BSI caused by *P. aeruginosa* is increasing. One of the studies reported that respiratory tract and central venous catheters were found to be the most frequent sources of BSI. Other risk factors include immuno-compromised patients in ICU, lung cancer, septic shock, pneumonia, having severe disease, delayed antimicrobial therapy, and multidrug resistance (70-72).

*Urinary tract infections*

Urinary Tract Infections (UTIs) are also another common type of acute and chronic infections caused by *P. aeruginosa*, they generally occur after catheterization, instrumentation, or surgery. Urinary tract catheterization is known to be a major cause of nosocomial acquired-UTI by *P. aeruginosa* as the pathogen utilizes catheters as a medium of bacteria entry resulting in attaching to catheter surface and biofilm formations (73-77).

*Skin and soft tissue infections*

Multidrug resistant *P. aeruginosa is* the most common cause of severe wound and burn infections and is associated with high morbidity and mortality rates worldwide. Various studies reported nosocomial outbreaks of the pathogen in surgical wounds resulting in post-operative wound infections (78-80). Some severe soft tissue infections have also been investigated which are associated with *P. aeruginosa *such as follicular dermatitis or folliculitis (a condition described as an itchy rash with a red base and white pustules), nail disease (onychosis) also known as green nail syndrome, paronychial infection (associated with prolonged exposure to moisture), onycholysis and onychomycosis in post-surgical patients, burn wound sepsis, pyoderma, dermatitis, and ecthyma gangrenosum. Mild skin infections have been reported in some previously healthy persons caused by *P. aeruginosa *adulteration in swimming pools, hot tubs, and other water sources (81-86).

*Eye infections*

*P. aeruginosa *is the main root of bacterial keratitis and it occurs in patients with several medical conditions such as pre-existing ocular diseases, post-ocular surgery, and patients using contact lenses. After adhesion, *P. aeruginosa* damages corneal epithelial cells and internalize rapidly. The contact lens may damage the epithelial surface of the cornea resulting in corneal keratitis in case of prolonged use or contamination of contact lens and improper handling or care by patients. Some studies reported that there is a rare occurrence of infections like endophthalmitis and neonatal ophthalmia in some patients caused by *P. aeruginosa *(87-91).

*Ear infections*

It is well known that *P. aeruginosa* is the most common cause of ear infections namely otitis externa (swimmer’s ear), which involves inflammation of external auditory occurring on prolonged exposure to moisture or associated with swimming in contaminated recreation pools and/or the insertion of foreign objects such as cotton buds, etc. Other types of infections caused by the pathogen are canal chronic supportive otitis media and malignant external otitis (92-94).

*Miscellaneous*

Other than above mentioned common infections, *P. aeruginosa *also contributes to some rare infections. The infection of the blood caused by any bacteria is called bacteremia and septicemia and the common symptoms observed in *Pseudomonas* infection of lungs and blood are fever, chilling, fatigue, muscle and joint pain, and cough with or without sputum accompanied by difficulty in breathing. *P. aeruginosa* also causes meningitis and brain abscess, infections related to the central nervous system, which are rare and secondary to neurosurgery or trauma. The pathogen also causes infections affecting bones and joints resulting in the development of several rare disease conditions such as steno-articular pyoarthrosis, vertebral osteomyelitis, symphysis pubis infection, osteochondritis of the foot, and chronic contiguous osteomyelitis. Rarely seen in drug addict patients, *P. aeruginosa* affects the heart leading to endocarditis (95-100).

***Virulence factors in P. aeruginosa***

The bacteria adhere to the host tissue with the help of pili, flagella, exo-enzymes, and exopolysaccharides. Colonization of this bacterial species is promoted by glycoprotein consisting of *N*-acetyl glucosamine (GlcNAc), N-acetylgalactosamine (GalNAc), D-mannose, L-fucose, and *N*-acetylneuraminic acid (NeuAc) sugar motifs (46). The various virulent factors which are responsible for the pathogenicity of *P. aeruginosa* are:

Protease, which causes ulceration and infections.

Exotoxin spread infections in the wounds of burn patients.

Phospholipase, which is associated with chronic pulmonary colonization.

Exotoxin A has also been shown to induce host cell death by apoptosis; it is an immunotoxin that targets tumor cells for anticancer therapy.

Lipases and phospholipases break down surfactant lipids and the phospholipids of host cell membranes.

The blue-green pigment pyocyanin gives *P. aeruginosa* colonies their distinct color and causes oxidative stress to the host, disrupting host catalase, and mitochondrial electron transport.

 Purified pyocyanin has been shown *in vitro* to induce apoptosis in neutrophils (101,102).

**Figure 2:**
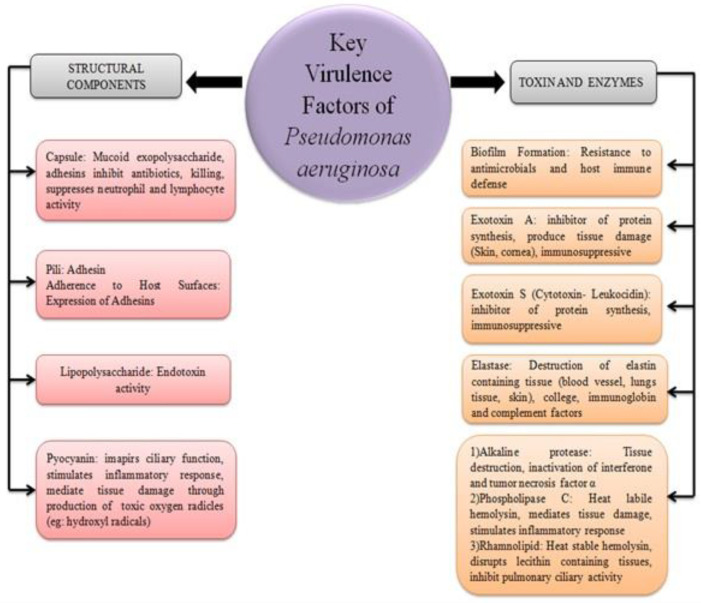
Schematic representation of key virulence factors of *Pseudomonas aeruginosa*

*P. aeruginosa *causes acute infections mainly in three steps, i.e., adhesion, invasion, and systemic spreading. It utilizes cell-associated and extracellular virulent factors to attack the host cell which causes damage to the host skin and reduces the efficiency of the immune system. In immunocompromised patients, the pathogen adheres to epithelial cells and utilizes sugar-binding proteins such as fimbriae (Polar, Type IV pili), flagella, and lectins (LecA and LecB) for the production of elastases, LasA, and LasB which exert cytotoxic effects on respiratory cells and promote bacterial adhesion to airway mucosa. These produced enzymes, hydrolase elastin, an essential protein of connective tissue that is considered to be an important factor of lung innate immunity (103-108). Also, *P. aeruginosa *facilitates the production of rhamnolipids and hemolytic phospholipases C responsible for the dissolution of phospholipids (phosphatidylcholine) present in the eukaryotic cell membrane and lungs. Moreover, the pathogen synthesizes the redox toxin pyocyanin which obstructs multiple mammalian cell functions such as cell respiration, metal-ion uptake, etc (105,109,110). After colonization at the site of infection, the same can spread the infection in the whole body through systemic circulation using the same virulence factors involved in adhesion and invasion steps leading to the development of biofilms (a heterogeneous structure consists of exopolysaccharide, rhamnolipids, extracellular DNA and proteins) at the colonized sites of host tissues with improved adhesion and stabilization, which causes the establishment of chronic infection and creates a physical barrier to several biocides, the immune system, UV light and antimicrobial agents (105). Moreover, the overall bacterial community formed in biofilm is not homogeneous. The cells present in the middle of the heterogeneous matrix are dormant and comparatively less metabolically active than the cells located on the surface due to low access to oxygen. Taking this fact into consideration, the effect of antibiotics becomes less effective as these agents can only kill pathogens with an active metabolism, for instance, cells on the surface of the biofilm. The bacterial cells attached to the inner layer of the biofilm remaining unaffected by antibiotics are then called persisters. As the concentration of antibiotics reaches sub-inhibitory levels, the persisters tend to switch their metabolic pathway on to repopulate the tissue causing the unmanageable infections which are very difficult to eradicate (111-113). Besides, patients with severe underlying diseases reducing physical (burn patients, mechanically ventilated patients) and/or immune defense mechanisms (neutropenia, AIDS patients) are at serious risk for the evolution of localized infections toward systemic disease, which is associated with dramatically elevated mortality. Just as varied as the clinical diseases caused by *P aeruginosa*, this typical nosocomial pathogen possesses and produces a large variety of both cell-associated and extracellular virulence factors. It is important to realize that the pathogenesis of *P. aeruginosa* is not related to a single virulence factor, but the precise and delicate interplay between different factors, leading from efficient colonization and biofilm formation to tissue necrosis, invasion, and dissemination through the vascular system, as well as activation of both local and systemic inflammatory responses. Extensive studies have shown that *P. aeruginosa *is armed with a large arsenal of virulence factors (described in the following paragraphs), enabling it to breach the human innate immune system, to intoxicate host cells, and to modulate human adaptive immune mechanisms, serving the goal of establishing a systemic infection or more localized chronic colonization (114, 115). In this review, we will discuss the various virulence determinants that have been suggested to play a role during the pathogenesis of *P. aeruginosa* infections. The various virulence factors produced by *P. aeruginosa* have been diagrammatically represented in Figure 2.

***Mechanism of antibiotic resistance development by P. aeruginosa***

Generally, the three major mechanisms of antibiotics resistance in *P. aeruginosa *can be classified into intrinsic resistance, extrinsic (acquired) resistance, and adaptive resistance.

*Intrinsic resistance*

The intrinsic resistance mechanism in *P. aeruginosa *involves restricted outer cell membrane permeability and expression of efflux pumps that expel antimicrobial agents out of the cell and also promote the production of antibiotics-inactivating enzymes (116). The four major mechanisms responsible for intrinsic resistance of *P. aeruginosa *towards antibiotics are 1) target mutation, 2) restrict cell wall uptake, 3) efflux pump, and 4) drug inactivation.

*Extrinsic or acquired resistance*

In this mechanism, bacteria attain resistance by mutational changes at the genetic level *via* horizontal gene transfer. The extrinsic mechanism significantly contributes to the development of multi-drug resistant pathogens leading to extreme difficulty in the eradication of microorganisms, which results in boosting cases of persistent infections (117, 118). A study reported that there are two ampG homologs in *P. aeruginosa *namely ampG (PA4393) and ampG1 (PA4218). ampG is only a functional protein and its inactivation by mutational change leads to a non-inducible and low-level β-lactamase expression (119).

*Adaptive antibiotic resistance*

This type of resistance mechanism is associated with increased ability of the pathogen for survival against antibiotics attack due to transient alterations in gene expressions in response to environmental stimuli and the mechanism gets reversed when the stimuli are removed. In *P. aeruginosa *to represent the adaptive resistance, the best-mentioned mechanisms are biofilm formation and development of persisters leading to persistent infections in CF patients (120, 121).

*• Biofilm formation*

Biofilms are specific and organized communities of cells under the control of signaling molecules, rather than random accumulations of cells resulting from cell division. These biological communities can be embedded in an extracellular matrix that is self-produced. Biofilms may help maintain the role of bacteria as pathogenic by evading host immune mechanisms, resisting antimicrobial treatment, and withstanding competitive pressure from other organisms. Consequently, biofilm-related infections are difficult to treat as they are less sensitive to anti-microbial agents. Biofilm production is also associated with a high level of antimicrobial resistance of the associated organisms (122-125).

*• Persistent cell-induced resistance*

This involves the formation of bacterial persister cells in the presence of high concentrations of antibiotics. Though, these persister cells (phenotypic variant) are not genetically resistant to antibiotics but are formed as a result of the heterogeneous response to the environment among the bacterial community which is genetically identical (115, 126-129).

**Biofilms of** ***P. aeruginosa***

**Figure 3:**
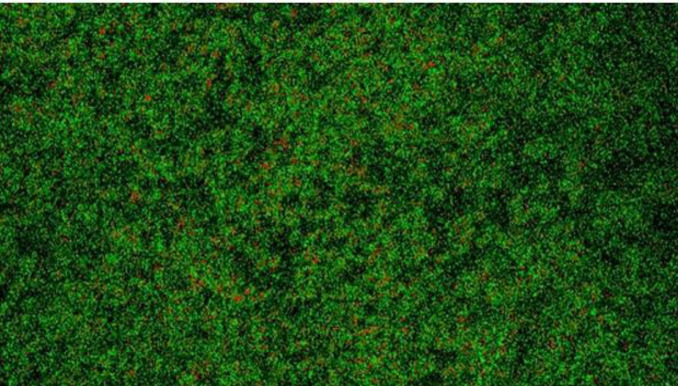
*Pseudomonas aeruginosa* biofilms confocal image (surface material: coverslip, taken by Dr Shaminder Singh using a Nikon A1 Confocal Laser Microscope System)

Biofilms are communities of microorganisms protected by a self-synthesized layer of complex polysaccharides, proteins, lipids, and extracellular DNA, collectively called the extracellular polymeric substance (130). Being in a biofilm, microbes are covered by a lot of advantages, including, but not limited to physical protection from the host immune system and antimicrobials/antibiotics, retention of water and tolerance to desiccation, nutrient sorption and storage, high extracellular enzymatic activity, adhesion to the infection site, and cell aggregation leading to coordination of virulence factor expression *via *QS (131-133). Particularly troubling to the medical field, it has been estimated that as much as 80.0% of all human bacterial infections are biofilm-associated, including more than 90.0% of all chronic wound infections (134, 135). Additionally, the biofilm mode of microbial life is responsible for up to a 1000-fold increase in antibiotic tolerance due to the physical impedance and enzymatic inactivation of the drugs, coupled with lowered metabolic rates in many biofilm-associated cells. Thus, biofilm infections are highly recalcitrant and are associated with chronic, non-healing infections (136, 137). Biofilms cause clinical problems of concern because they increase resistance to antifungal therapy; one hypothesis of the mechanism of biofilm resistance is the presence of the matrix that restricts the penetration of drugs through the formation of a diffusion barrier and only the most superficial layers are in contact with lethal doses of antibiotics (138). *P. aeruginosa *can form a biofilm in various environments. Biofilms have been known to have a rather complex structure with (to a certain level) differentiated bacterial populations and increased resistance against hostile environmental factors, including host immune mechanisms and treatments such as antibiotics. Evidence indicates that *P. aeruginosa *forms a biofilm in CF lungs where the bacterium lives in an anaerobic environment, as opposed to the aerobic biofilm formed in laboratory conditions. The biofilm mode of growth is recognized as an important bacterial trait that is relevant to infections (122,139). The biofilm formed by *P. aeruginosa *is shown in Figure 3.

Many infections involve the formation of bacterial biofilms, which are bacterial communities that settle and proliferate on surfaces and are covered by exopolymers. Once established, biofilms are difficult to eradicate and become a source of secondary infection. The dose of antibiotics needed in this situation will often exceed the highest deliverable dose, which makes efficient treatment impossible (140).

**Role of quorum sensing in** ***P. aeruginosa*** **virulence**

Several new approaches are being actively developed for curbing *P. aeruginosa *infections over conventional antibiotic chemotherapy in clinical practice. Some of them are based on QS and biofilm inhibition, which is characterized under anti-virulence strategies.

***Quorum sensing mechanism***

QS phenomenon involves microbial behaviors or responses that are governed by microbial cell density. This mechanism occurs in both Gram-positive as well as Gram-negative bacteria. Such community behaviors are usually determined by secreting signaling molecules, so-called auto-inducers (AIs), accumulation of which is a measure of cell density and nutrient concentration such as iron and phosphate. QS has a pivotal role in biofilms of all kinds (141). Bacteria produce and release small diffusible molecules, usually termed signals, which have two main consequences. First, the uptake of these molecules into cells regulates (auto-induction) a whole variety of behaviors, including the production of a range of exofactors that are released from the cells to aid growth, motility, and/or biofilm formation. Second, the uptake of these molecules also leads to an increase in the production of the signal molecule itself (auto-regulation). The production of these signals or autoinducer molecules, therefore, leads to positive feedback at high cell densities, which results in a considerable increase in the production of signal and QS controlled factors (Figure 2). The hypothesis here is that producing certain extracellular factors is most beneficial at high cell densities and that QS provides a mechanism that allows cells to increase the production of extracellular factors at high cell density (142-144).

**Figure 4:**
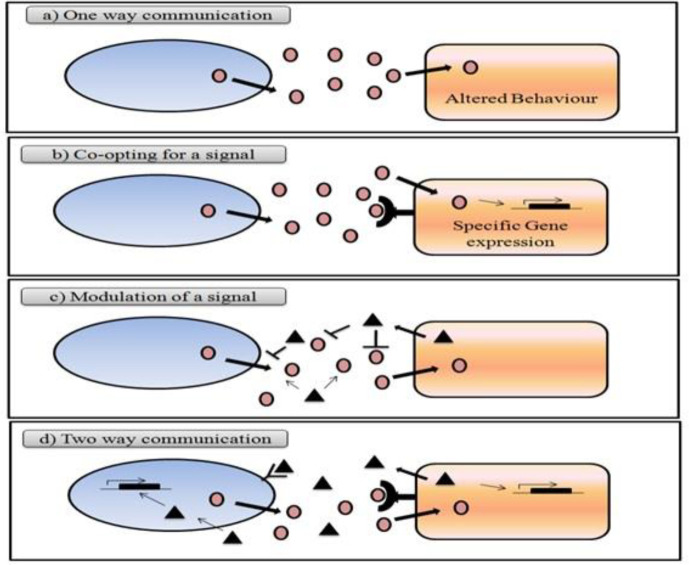
Examples of uni and bi-directional signaling interactions

In many cases, autoinducers and other molecules are not only responsible for same-species communication but also for the more complex interspecies cross-talk. The diversity of inter-kingdom signaling occurring in a myriad of environments has been classified into four categories:

(1) One-way sensing: one organism senses and responds to a diffusible signal produced by a second organism;

(2) Co-opting for a signal: one organism uses the signal produced by another to regulate its gene expression;

(3) Modulation of a signal: one organism alters the production or stability of a signal from another organism; and 

(4) Two-way communication: multiple signals are exchanged between organisms (145) as shown in Figure 4.

***Quorum sensing in P. aeruginosa***

**Figure 5:**
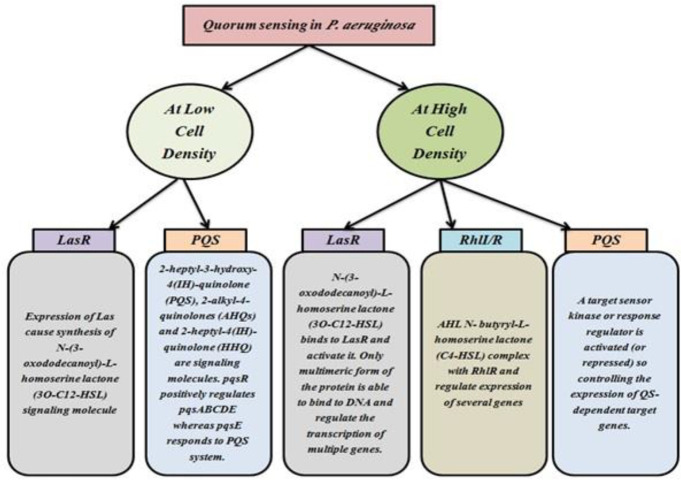
Different quorum sensing systems in *Pseudomonas aeruginosa*

**Figure 6:**
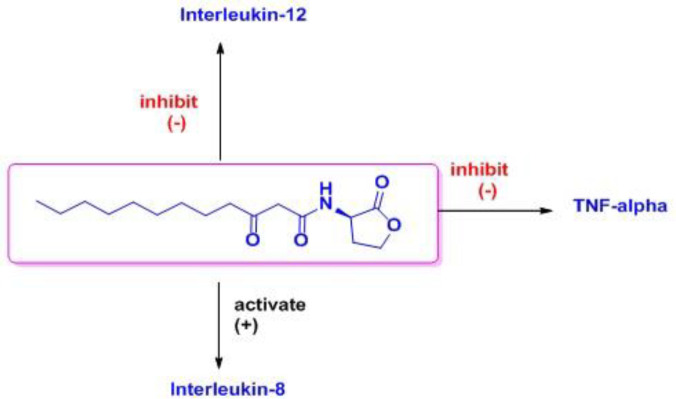
Diagrammatic representation of the effect of N-(3-oxododecanoyl)-L-homoserine lactone (3O-C12-HSL) signaling molecule on the LasR system

**Figure 7:**
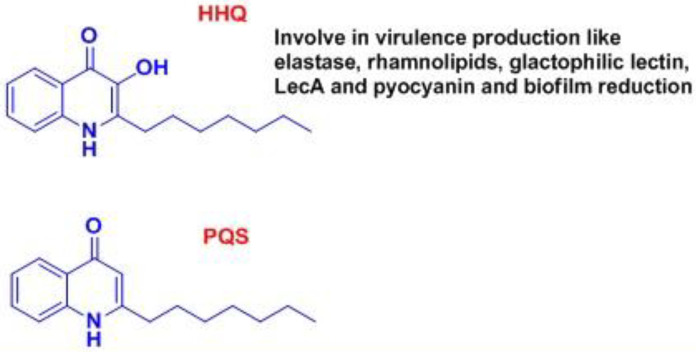
Representation of the effects of Pqs quorum sensing in virulence production

The behavior of *P. aeruginosa* is monitored by a complex regulatory mechanism called Quorum sensing (QS) in acute and chronic infections (12). The co-ordination of specific gene expression in the community involves the interaction of diffused molecular signals. The quorum-sensing system depends on 3 basic principles in the bacterium. First, the production of AIs also called signaling molecules by the bacterial population. At low cell density, these signaling molecules diffuse away and therefore are present at concentrations below the threshold required for detection. At high cell density, the cumulative production of signaling molecules results in high concentration locally facilitating detection and response. Second, these AIs are detected by the receptors present in the cell (cytoplasm or membrane). Third, the detection of AIs facilitates AI production to potentiate the expression of genes. This feed-forward auto-induction loop promotes the development of the population (146-148). In *P. aeruginosa*, the quorum-sensing circuit is controlled by the expression of gene systems *viz.* four different QS channels interlinked to each other for disseminating virulence, biofilm production, and synthesis of signal molecules. The channels are *las*, *Rhl*, *Iqs*, and *Pqs* where these systems employ transcriptional regulators such as LasR, RhlR, IqsR, and PqsR, respectively (also known as Multiple virulence factor regulator, MvfR). This MvfR binds to specific AIs to aggravate the expression of selected genes to cause virulence. The expression of different QS systems took place in response to the varying levels of cell density (Figure 4). Furthermore, the *las, rhl *and *Pqs *based systems coordinate biofilm formation. The *las* system utilizes N-(3-oxododecanoyl)-L homoserine lactone (3-oxo-C12-HSL) as a signal molecule that induces the expression of LasA and LasB elastases, alkaline protease, MvfR, RhlR, IqsR, and the cognate synthetase LasI. The Rhl system uses a molecule of N-butanoyl-L-homoserine lactone (C4-HSL) as an auto-inducer (belonging to an acyl-homoserine lactone (AHL)) which facilitates the synthesis of rhamnolipids, LasB elastase, pyocyanin, hydrogen cyanide RhlI (related signal molecule biosynthetic protein), and down-regulation of mvfR. This chemical triggers the production of inflammatory mediators. However, in the case of chronic infections, the *rhl* system is expressed and maintained for a longer duration (149-151). The recently discovered* Iqs* system employs 2-(2-hydroxyphenyl)-thiazole-4-carbaldehyde (IQS), which is supposed to regulate the Pqs system (152). The *Pqs* system makes use of quinolone signals (QS) molecule which helps in the synthesis of pyocyanin, hydrogen cyanide, as well as LecA lectin, the enzyme required for Pqs biosynthesis and the expression of RhlR and LasR. Along with the production of acyl-homoserine lactone as QS signals in *P. aeruginosa*, the other class of autoinducers is 4-hydroxy 2-alkyl quinolones (HAQs) and derivatives of 4-hydroxy-2-heptylquinoline (HHQ), including di-hydroxy derivatives like 2-heptyl-3,4-dihydroxyquinoline (152-155). All the different types of QS mechanisms such as *lasR*, *rhls*, and *Pqs* have been depicted in Figure 5, 6, and 7, respectively. 

***Quorum sensing inhibition as an anti-virulence strategy***

QS is known to be an extremely important mechanism in the regulation of virulence factors as well as the formation of biofilms so it has become a potential target to minimize drug resistance during the treatment. Various *in vivo* studies showed that strains lacking in the expression of transcriptional regulators or auto-inducer (AI) biosynthetic pathway give rise to lower mortality of mice as compared with the animals treated with wild type of *P. aeruginosa. *Three different approaches can be considered while designing QSIs such as signal molecule inactivation, inhibition of AI syntheses, and interference with transcriptional regulators (156-158). Various studies have been conducted targeting QS inhibition as an anti-virulence strategy against various resistant pathogens including *P. aeruginosa*. For instance, a study (2015) shows that novel *N*,*N*-disubstituted biguanides were found to have QS inhibition activity against *Chromobacterium violaceum *(159). In another study by Singh S,* et al.* (2016), phenolic compounds from ginger rhizomes exhibited a QS inhibitory activity against *C. violaceum* and *P. aeruginosa *(160). Furthermore, in a study based on *in silico* docking, ADME, and toxicity, aryl glycoxamide derivatives were found to have substantial potential to develop as anti-virulent agents to inhibit QS in *P. aeruginosa *and* E. coli *(161). In another report, molecular docking studies were carried out for novel 1,8- Naphthyridine derivatives and showed moderate to good anti-bacterial activity tested against various strains such as *P. aeruginosa*, *E. coli*, *Staphylococcus aureus,* and *Bacillus subtilis *(162). Thus, these findings make a basis to consider the QS mechanism as a potential target for anti-virulence strategy.

**Targeting quorum sensing proteins is a remedial solution to multi-drug resistant strains**

***Autoinducers involved in quorum sensing of P. aeruginosa***

QS is a process in which both Gram-positive and Gram-negative bacteria monitor their species, modulate intra- and interspecies cell to cell communication, control expression of specific genes in response to fluctuation in cell population density and regulate diverse physiological functions by releasing chemical signaling molecules known as autoinducers (AI), example N-acyl-homoserine lactone (AHL). Table 1 (entry 1-9) shows a different kind of AI released during QS (163, 164).

***Chemical classes of compounds that inhibit quorum sensing in P. aeruginosa***

*P. aeruginosa* contains an MvfR QS system. These systems can be targeted to attenuate the virulence of *P. aeruginosa*. Some research groups have found that the *P. aeruginosa* mutants lacking the *las* gene are a-virulent type and unable to cause pneumonia. Rhl mediated QS inhibition includes the *rhlR* encoded putative transcriptional activator, RhlR, and *rhlI* encoded putative AI synthase, RhlI. A second *P. aeruginosa* Auto-inducer (PAI-2), N-butyryl homoserine lactone, was shown to re-store rhamnolipids production in a *P. aeruginosarhlI* mutant and also require *rhlI* for its synthesis. Some compounds also have been found that can act as inhibitors of both Las and Rhl mediated QS and could be beneficial to curb the pathogenesis of *P. aeruginosa *(165). In contrast, the Pqs system is associated with QS through quinolone signaling molecules and can be targeted by QSIs to inhibit bacterial virulence (166, 167). In *P. aeruginosa*, both QS and biofilms are impacted by the surrounding environment representing these complex communities as a challenge (168). A variety of potent chemical compounds can be utilized to inhibit the process of QS and thereby reducing the QS-mediated biofilm formation in *P. aeruginosa*. In this quest, various reported synthetic compounds have been found which act on attenuating the *P. aeruginosa* virulence by targeting various QS mediated systems as represented in Table 2.

**Concluding remarks**

It is well-established that the pathogenic microbial strains possess an enhanced ability to adapt and develop a mechanism against the chemical compound that could impair its sustainability. The overuse of antibiotics increases the chances of development of resistant strains. This is especially true for the opportunistic pathogen *P. aeruginosa* and its inherent capability to transform into a multidrug-resistant phenotype. However, this pathogen can rapidly develop resistance to multiple classes of antibiotics during patient treatment. The chromosomal protein AmpG, the outer membrane porin OprD, and the multitude of efflux pumps are particularly responsible for this challenging therapeutic regime, and the discussion presented in this review highlights complex mechanisms and pathways by which *P. aeruginosa* regulates and/or co-regulates their expression. In the lack of a diminished antibiotic development pipeline towards antimicrobial therapeutics, we must look for novel strategies to combat the threat of antibacterial resistance. To solve this issue, an alternative strategy that involves the development of new active agents that are capable of targeting bacterial virulence besides its growth has to be devised. In this context, research for anti-QS has been largely explored during the last two decades to propose new alternatives to struggle against bacterial infection with limited selective pressure. The present study highlights the importance of QS in up-regulation of efflux pump genes for escaping from antibiotic attack. However, the scientific community has to admit the importance of QS in the development of bacterial resistance, and concealed pathways have to be explored for investigating the role of QSI in order to develop anti-QS therapeutics. 

**Table 1:** Different analogs of Acyl Homoserine Lactone (AHL)

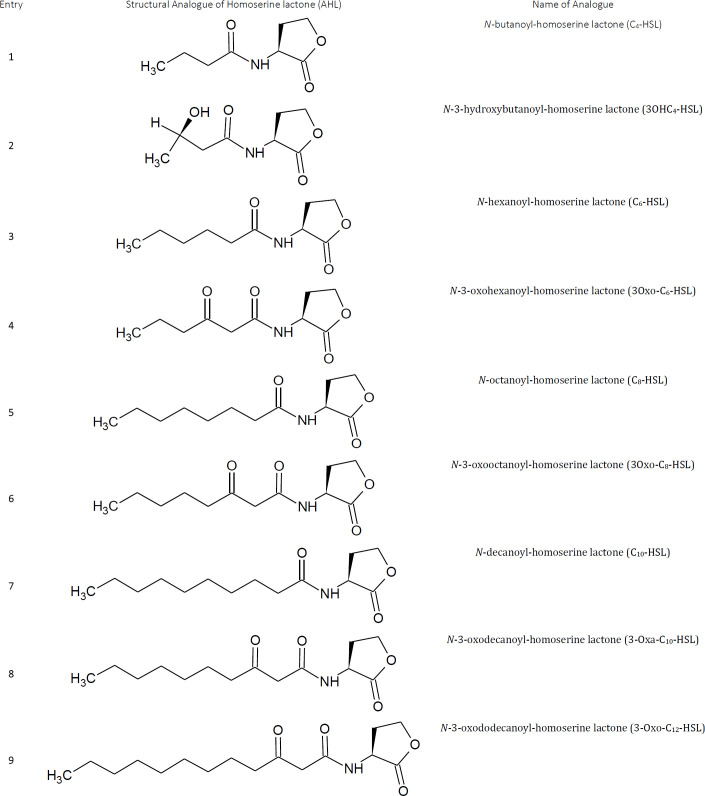

**Table 2:** Chemical classes reported as quorum sensing inhibitors that act via different mechanisms against *Pseudomonas aeruginosa*